# Draft genome sequence of three antibiotic-resistant *Klebsiella* spp. isolates from wastewater samples

**DOI:** 10.1128/mra.00281-25

**Published:** 2025-05-07

**Authors:** Marta Lois, Sergio Rodríguez, Jesús L. Romalde

**Affiliations:** 1 CRETUS, Department of Microbiology and Parasitology, CIBUS-Faculty of Biology, Universidade de Santiago de Compostela 16780, Santiago de Compostela, Spain; 2 iARCUS, Department of Microbiology and Parasitology, CIBUS-Faculty of Biology, Universidade de Santiago de Compostela 16780, Santiago de Compostela, Spain; DOE Joint Genome Institute, Berkeley, California, USA

**Keywords:** *Klebsiella michiganensis*, *Klebsiella pneumoniae*, wastewater, antibiotic resistance, fluoroquinolones, β-lactams

## Abstract

The draft genome sequences of two *Klebsiella michiganensis* and one *Klebsiella pneumoniae* subsp. *pneumoniae* strains isolated from wastewater in Galicia (NW Spain) are reported. Genomic analysis revealed that all *Klebsiella* strains harbor multiple genes associated with resistance to fluoroquinolones, β-lactams, and trimethoprim, among other drugs.

## ANNOUNCEMENT

Enteric bacteria constitute a significant portion of the bacterial pathogens present in wastewater, including genera such as *Escherichia*, *Shigella*, *Klebsiella*, and *Salmonella*. These bacteria inhabit the human intestinal tract and can cause gastrointestinal infections (1, 2). Among them, *Klebsiella pneumoniae* and *Klebsiella michiganensis* are more commonly associated with opportunistic infections, with the former being a major concern in hospital settings (3, 4).

In this work, two antibiotic-resistant *Klebsiella michiganensis* (a14 and a16) strains and one *Klebsiella pneumoniae* subsp. *pneumoniae* (a4) strain were isolated and characterized from sewage samples during October and November 2022 in Galicia (North-West Spain; 42.20599062 N 8.73005254 W) in a decentralized system as previously described (5) using Klebsiella Chromogenic Agar supplemented separately with ciprofloxacin (1 µg mL^-1^), trimethoprim (100 µg mL^-1^), and sulfamethoxazole (7 µg mL^-1^). Preliminary identification of the isolates was performed by 16S rRNA gene sequencing, as previously described (6). Strain a14 was isolated from the inflow of black waters, and strains a4 and a16 from the inflow of gray waters. Multiresistance of the strains was confirmed in antibiogram assays on Mueller-Hinton agar using the disk diffusion method (7) under the Clinical and Laboratory Standards Institute guidelines (8) (Table 1).

**TABLE 1:** Assembly statistics for the genome sequences reported in this study

Genome	GenBank accession no.	SRA accession no.	Number of reads	Avg coverage (×)	Total contig length	Number of contigs	*N*_50_	Largest contig size (nt)	G + C (%)	CDS number	Total genes	Protein-coding genes	rRNA genes	tRNA genes	Resistance to^a^
a4	JBJUWH00000000	SRR31677190	4,186,818	34	5,510,686	120	98,362	268,154	57.60	5,343	5,408	5,095	4	54	AML KF S
a14	JBHKAI000000000	SRR30781977	4,615,045	31	6,207,592	180	74,192	213,620	56.50	5,972	6,034	5,834	2	56	AML E KF RL S UB W
a16	JBHKAH000000000	SRR30870652	5,216,019	31	6,248,724	203	67,434	194,603	56.20	6,041	6,102	5,888	3	54	AML E KF RL S UB W

^a^ Results obtained by disk diffusion method. Antibiotics (µg/disk): AML, amoxicillin (25); E, erythromycin (5); KF, cephalotin (30); RL, sulfamethoxazole (25); S, streptomycin (10); UB, flumequine (30); and W, trimethoprim (5).

Genomic DNA was isolated from 24 h pure cultures grown on trypticase soy agar at 37°C, using the DNeasy Blood & Tissue Kit (Qiagen, Mississauga, ON, Canada). The DNA was sequenced at Sistemas Genómicos (SGLabs, Valencia, Spain). Sequencing libraries were prepared with the Nextera XT DNA Library Prep Kit and sequenced with the MiSeq instrument (Illumina) using paired-end read technology (2 × 150). The quality of the raw data was checked using FASTQC version 0.9.1 (9). Low-quality reads and sequencing primers were removed, and final quality filtering was carried out using Trimmomatic version 0.39 (10). High-quality reads were assembled with Megahit (11). The publicly available genome was annotated using the NCBI Prokaryotic Genome Annotation Pipeline version 6.7 (12). Resistance genes were evaluated using ResFinder version 4.5.0. (https://cge.food.dtu.dk/services/ResFinder/) and the Comprehensive Antibiotic Resistance Database (version 3.2.9; https://card.mcmaster.ca/analyze/rgi), with a minimum identity of 95%. Average nucleotide identity (ANI) among the different *Klebsiella* genome assemblies was calculated by PYANI 0.2.11 (13). Default parameters were used for all software unless otherwise specified.

Strains a14 and a16 yielded ANI values of 99.21% and 99.22%, respectively, with the reference strain *Klebsiella michiganensis* THO-011 (GenBank accession number GCA_015139575.1), whereas strain a4 showed an ANI of 99.19% with the reference strain *Klebsiella pneumoniae* subsp. *pneumoniae* HS11286 (GCA_000240185.2). The genome sizes and G + C content of strains were 6.2 Mb and 56.5% for a14, 6.2 Mb and 56.2% for a16, and 5.5 Mb and 57.6% for a4 (Table 1). Detailed statistical metrics for each genome sequence are given in Table 1. Among others, several genes involved in resistance to fluoroquinolones (*rsm*A, *emr*R, and *ade*F), β-lactams (CRP, *omp*A), aminoglycosides (*bae*R), and polypeptidic antibiotics (*van*G and *ept*B) were found in all *Klebsiella* genomes. *Klebsiella pneumoniae* subsp. *pneumoniae* strain a4 also harbored other AMR genes, such as the efflux pump genes *oqxA* and *oqxB*, and the beta-lactamase genes *bla*_SHV-26_, *bla*_SHV-98_, *bla*_SHV-78_, *bla*_SHV-145_, *bla*_SHV-179_, *bla*_SHV-194_, and *bla*_SHV-199_. Moreover, the *bla*_OXY-1-3_ gene was found in *Klebsiella michiganensis* strain a14.

## Data Availability

The whole-genome sequencing project has been deposited at NCBI under BioProject accession number PRJNA1113845 and BioSamples SAMN45227409 (a4), SAMN43828072 (a14), and SAMN43828073 (a16). Raw sequences have been deposited into the Sequencing Read Archive under accession numbers SRR31677190 (a4), SRR30781977 (a14), and SRR30870652 (a16). The assembled genome sequences were deposited at NCBI under accession numbers JBJUWH000000000 (a4), JBHKAI000000000 (a14), and JBHKAH000000000 (a16).
